# Genomes of *Wiebesia* Fig Wasps Reveal the Adaptation and Codiversification in the Fig–Fig Wasp Mutualism

**DOI:** 10.1093/gbe/evag080

**Published:** 2026-04-18

**Authors:** Bai-Wei Lo, Hsin-Fu Lin, Siu-Wah Kong, Wen-Jer Wu, Yi-Lun Peng, Selina Cai-Ling Wang, Xuemei Lu, Hurng-Yi Wang

**Affiliations:** Research Group of Development and Disease, Max Planck Institute for Molecular Genetics, Berlin, Germany; Institute of Ecology and Evolutionary Biology, National Taiwan University, Taipei, Taiwan; Graduate Institute of Clinical Medicine, College of Medicine, National Taiwan University, Taipei, Taiwan; Department of Entomology, National Taiwan University, Taipei, Taiwan; Department of Entomology, National Taiwan University, Taipei, Taiwan; Institute of Ecology and Evolutionary Biology, National Taiwan University, Taipei, Taiwan; Department of Entomology, National Taiwan University, Taipei, Taiwan; Beijing Institute of Genomics, Chinese Academy of Sciences, Beijing, China; Kunming Institute of Zoology, Chinese Academy of Sciences, Kunming, China; Institute of Ecology and Evolutionary Biology, National Taiwan University, Taipei, Taiwan; Graduate Institute of Clinical Medicine, College of Medicine, National Taiwan University, Taipei, Taiwan; Department of Entomology, National Taiwan University, Taipei, Taiwan

**Keywords:** *Ficus pumila* var. *awkeotsang*, *Ficus pumila* var. *pumila*, codiversification, coevolution, olfactory receptor, *Wiebesia*

## Abstract

Figs and fig wasps represent one of the most intimate examples of plant–pollinator coevolution. As figs diversified into geographically isolated populations, both figs and fig wasps underwent selective pressures driven by local adaptation and coevolution. *Ficus pumila* comprises two ecologically distinct varieties: the creeping fig (*F. pumila* var. *pumila*), which is widely distributed across the lowlands of East Asia, and the jelly fig (*F. pumila* var. *awkeotsang*), endemic to Taiwan and found at mid-elevations. To elucidate how codiversification with fig hosts influences the evolutionary trajectories of fig wasps, we analyzed the genomes of *Wiebesia* sp. 2 and sp. 3, the respective pollinators of creeping fig and jelly fig. Our demographic analysis indicates that vicariance during the Last Glacial Period facilitated ecological differentiation between these two fig–fig wasp pairs. Through comparative and population genomic analyses, we identified selection signals linked to habitat adaptation, with evolutionary rates corresponding to the life history traits of their host figs. Variations in host preference behavior, chemosensory gene expression, and adaptive duplications in olfactory receptors highlight potential mechanisms for adaptation to host floral scents. These findings collectively underscore how the obligate mutualism between figs and their pollinating wasps allows the ecological traits and habitat preferences of fig hosts to shape the evolutionary pathways of their pollinators, leaving distinct molecular imprints in the fig wasp genomes. This study demonstrates the capacity of tightly intertwined life cycles between plants and pollinators to drive adaptation and diversification.

Significance Codiversification among plants and their pollinators is a pervasive evolutionary phenomenon, yet the genomic mechanisms driving these interactions and their subsequent effects on both parties remain inadequately understood. This study leverages the classic coevolutionary relationship between figs and pollinating wasps to examine the genomics and behavior of two *Wiebesia* species that have codiverged alongside their ecologically distinct *Ficus pumila* hosts. Our research unveils key molecular mechanisms that underpin coadaptations, shedding light on the genomic differentiations that facilitate plant–pollinator codiversification. These insights enhance our understanding of how complex mutualistic relationships affect diversification at the genomic level.

## Introduction

The interaction between flowering plants and their insect pollinators has contributed to the immense biodiversity of both groups (Dodd et al. 1999; Grimaldi 1999; Sargent 2004; Kay and Sargent 2009; Cruaud et al. 2012). Despite the prevalence of these interactions, our understanding of the genetic processes involved in codivergence at the microevolutionary scale—the initial step in generating biodiversity—remains limited (Hembry et al. 2014; Medina et al. 2022). One challenge in studying codiversification is the complexity of organisms’ interactions with their environments, making it difficult to disentangle the selective pressures from multiple biotic and abiotic sources (Althoff et al. 2014).

The mutualism between pollinating fig wasps (Hymenoptera: Agaonidae) and figs (Moraceae: *Ficus*) offers an exceptional system for exploring coevolution (Janzen 1979; Weiblen 2004; Herre et al. 2008). Figs have evolved peculiar, fruit-like inflorescences called syconia (singular: syconium) that house their flowers. The figs are exclusively pollinated by fig wasps. In return, the short-styled flowers (gall flowers) in syconia provide food and shelter for fig wasp larvae. Fig wasps spend almost their entire life cycle within the syconia. The pollen-bearing female wasps only leave the syconia after mating and have just a few days to search for another receptive syconium. Thus, figs and fig wasps share a common abiotic environment, while the biotic environment encountered by the wasps is largely restricted to the community within the fig syconia.

The female fig wasps are attracted to the unique floral volatile compounds of their associated hosts (Ware et al. 1993). Diversification of host floral scents affects their associated fig wasps’ preference behavior and can contribute to prezygotic isolation barriers (Ware et al. 1993; Grison-Pigé et al. 2002; Cornille et al. 2012; Souto-Vilarós et al. 2018). Recognition of scent is thus key to the specificity of the mutualism. The tightly bounded life cycles between figs and pollinating wasps suggest that ecological traits, such as life history, habitat preference, and floral scents of fig hosts, should have profound impacts on the fundamental evolutionary process of their associated pollinator, with associated molecular signatures in the genomes of pollinators (Yoder 2016).

**Fig. 1.**
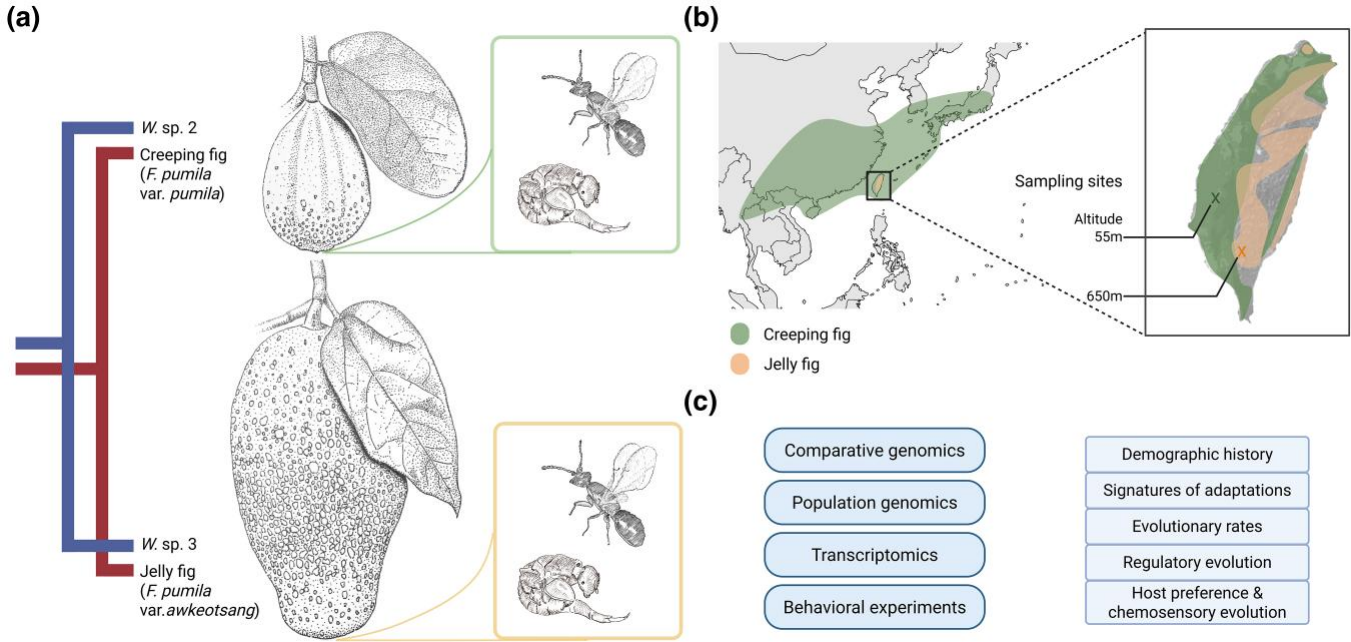
Research system. a) The cophylogeny of the studied *Wiebesia* spp. and their *F. pumila* hosts (Wang et al. 2013). Branchlets with syconia of the two fig varieties and their respective fig wasps were illustrated by Bai-Wei Lo. b) The presumed distribution of *F. pumila* varieties and the locations where the fig wasp genomic sequencing samples were collected. Geographical distribution of the two *Ficus pumila* varieties was modified from GBIF.org (2024). c) A summary of the experiments and analyses conducted. Created with BioRender.com/2wnj2oc.

Recent advances in genomic studies on nonmodel organisms have illuminated the coevolutionary dynamics between figs and fig wasps (Zhang et al. 2020; Wang et al. 2021). However, few studies have examined how differentiating populations are influenced by divergent coevolutionary pressures (Satler et al. 2019). Consequently, genomic investigations focused on closely related lineages are crucial for understanding the process of fig–fig wasp codiversification. The varieties of *Ficus pumila* L. and their associated pollinator wasps present ideal candidates for such studies. *F. pumila* is a functionally dioecious species that consists of two ecologically differentiated varieties. *F. pumila* L. var. *pumila*, known as the creeping fig, is widely distributed in the lowlands of East Asia, while *F. pumila* var. *awkeotsang* (Makino) Corner, or jelly fig, is found in the mid-elevations of Taiwan (Fig. 1a and b). These two varieties differ in life history traits, including syconium size and phenology (Fig. 1a) (Hsieh et al. 1993). Male creeping figs undergo two to four flowering cycles annually, whereas male jelly figs typically have one or two (Shih 1988 ; Ho et al. 1998). The varieties also differ in their floral volatile compound compositions (Chen and Wu 2010; Chen et al. 2016).

The ecology of *F. pumila* pollinators has been less thoroughly studied. Originally classified as a single species, *Wiebesia pumilae*, they have since been split into three species based on genetic evidence (Chen et al. 2012). *Wiebesia* sp. 1 and its southern sibling, *W.* sp. 2, pollinate the creeping fig, whereas *Wiebesia* sp. 3 pollinates the jelly fig. A pioneering genomic study on creeping fig and *W.* sp. 2 from Mainland East Asia has provided important insights into chemosensory coevolution in this pollination system (Wang et al. 2021). However, the biogeographic history and genomic mechanisms underpinning the codiversification between these fig wasps and fig varieties are not yet understood.

In this study, we present the genomic sequences of *W.* sp. 2 and *W.* sp. 3 from Taiwan, the pollinators of the creeping fig and jelly fig, respectively. These genomes offer comprehensive insights into the genomic processes of codiversification. We first reconstruct the demographic history to explain the niche partitioning of the two species. We then explore the genomic signatures of adaptations to different niches. Through behavioral experiments and transcriptomics, we examine the evolution of host preference in these closely related wasps. Finally, by integrating olfactory receptor (OR) annotations from other fig wasps, we investigate the consequences and potential genetic basis of host specificity in fig–fig wasp coevolution.

## Results

### Genome Sequencing, Assembly, and Annotation

We de novo assembled the genome of *Wiebesia* sp. 3 using PacBio long reads generated from genomic DNA extracted from 30 pooled males collected from a single syconium (Table S1) and applied Blobtools to remove possible contaminations (Laetsch and Blaxter 2017). Subsequent polishing of the draft genome using both long and short reads yielded a final genome of 323.8 Mbp (Table 1). It comprises 230 contigs (N50 = 18.6 Mbp), with a GC content of 30.16% and no sign of residual contamination (Fig. S1). The complete BUSCO score of *W.* sp. 3 genome is 93.7%. Ninety-seven percent of the genome sequences were assembled according to kmer estimates. The assembly statistics and BUSCO scores together suggest that this genome is highly contiguous and comparable to other fig wasp genomes (Xiao et al. 2013; Zhang et al. 2020; Wang et al. 2021; Chen et al 2022; Zhao et al 2025) (Table S2 and Fig. S2). Additionally, we obtained 80 × short reads from a *W.* sp. 2 population in Taiwan and performed a reference-based assembly.

**Table 1:** Assembly statistics of two *Wiebesia* species

	*Wiebesia* sp. *3*	*Wiebesia* sp. 2
Assembly size	323.8M	−^a^
Contigs	230	
GC (%)	30.16	
N50	18,609,986	
N75	6,255,745	
BUSCO completeness (genome)	93.7%	93.3%
Number of protein-coding genes	10,072	10,037
Average cds length (bp)	1,746	1757
Average exon number per gene	6.99	7.02
BUSCO completeness (annotation)	95.5%	95.7%

^a^Reference-based assembly.

We independently annotated the two genomes employing the same protocol. Approximately 10% of repetitive sequences were identified in the genome (Table S3). Using the EVidenceModeler pipeline (Haas et al. 2008), 13,342 and 13,684 protein-coding genes of *W.* sp. 3 and *W.* sp. 2 were annotated, respectively (Table S4). After manual curation, 10,072 and 10,037 genes were left in the gene sets (Table 1). Despite reduction in gene numbers, BUSCO values of the two species’ gene sets after manual curation improved from 93.0% to 95.5% in *W.* sp. 3 and from 92.8% to 95.7% in *W.* sp. 2. As a result, these annotations are among the most complete of all available fig wasp genomes (Table S2). Functional annotation of protein-coding genes reported that 95% of the genes have at least one match hit against public datasets (Fig. S3).

### Species Divergence Corresponds to the Last Glacial Period

**Fig. 2.**
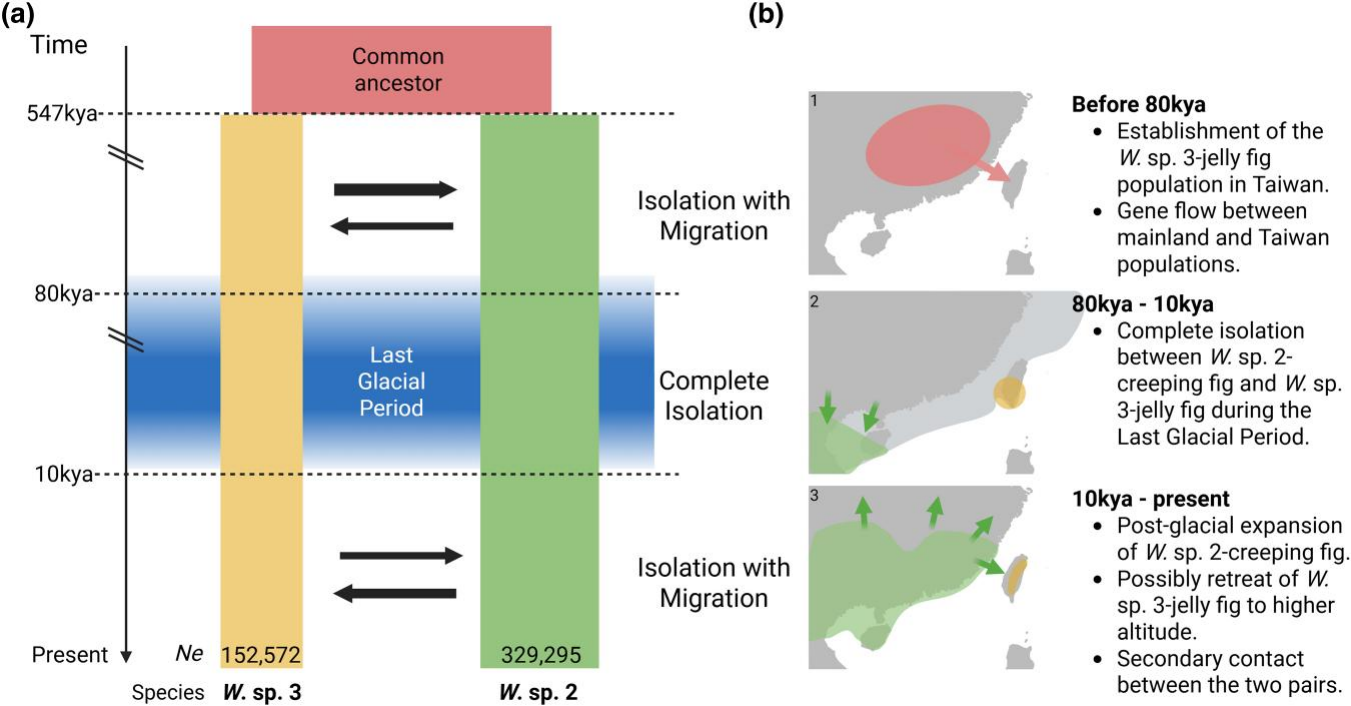
Demographic history of the two *Wiebesia* fig wasp species. a) The demographic model inferred by fastsimcoal2 shows a time of complete isolation that largely overlaps with the Last Glacial Period. The arrows in the middle represent gene flow between species. b) Proposed scenarios corresponding to each time segment based on current genetic and ecological evidence. The distribution of *W.* sp. 2 between 80 and 10 kya in b2 was based on the warm temperate evergreen forest biome predicted in the Last Glacial Maximum (Harrison et al. 2001). In present day, this biome largely overlaps with the distribution of *W.* sp. 2 (Chen et al. 2012), which is illustrated in b3. Created with BioRender.com/q14i636.

Both creeping fig and jelly fig have large-sized male syconia with thousands of gall flowers, which generally require 4 to 20 foundresses per syconium for successful pollination (Chen et al. 2002). We can therefore use the diversity of these foundresses as samples to estimate population genomic parameters. The genomic DNA libraries were constructed from pooled male individuals of a single syconium from the respective species. Because fig wasps are haplodiploid, this means that the libraries reflect the genetic diversity of the multiple foundresses. We applied the PoolParty pipeline (Micheletti and Narum 2018) to estimate genome-wide population genomic parameters. Nucleotide diversity was 0.13% and 0.24% for *W.* sp. 3 and *W.* sp. 2, respectively. We then used fastsimcoal2 (Excoffier et al. 2021) to simulate and evaluate different demographic models (Table S5). Among all models tested, the most supported scenario is the one considering both early and recent gene flow between *W.* sp. 3 and *W.* sp. 2 (Fig. 2; Table S5). In this scenario, *W.* sp. 3 and *W.* sp. 2 diverged during the Middle Pleistocene, about 547,000 years ago [95% confidence interval {CI}: 178,825 to 645,899], followed by subsequent gene flow, predominantly from *W.* sp. 3 to *W.* sp. 2. Gene flow ceased from ca. 80,000 (95% CI: 26,390 to 168,648) until 10,000 (95% CI: 5,514 to 12,687) years ago. Notably, this complete isolation period overlaps with the Last Glacial Period (115,000 to 11,700 years ago; Dansgaard et al. 1993). Afterward, gene flow was reestablished, with more prominent migration from *W.* sp. 2 to *W.* sp. 3.

### Evolutionary Rate Differences and Metabolic-Associated Adaptations

We compared the evolutionary rates between *W.* sp. 2 and *W.* sp. 3. The nonsynonymous substitutions (dN) and the synonymous substitutions (dS) of the *W.* sp. 3 lineage were 0.88 × 10^−3^ and 5.89 × 10^−3^, respectively. These estimates are significantly lower (*P* < 10^−10^) than those (dN, 1.15 × 10^−3^; dS, 7.05 × 10^−3^) of *W.* sp. 2 (Table 2). We also compared the evolutionary rates of different *W.* sp. 2 populations across the Taiwan Strait using a published *W.* sp. 2 genome from mainland China (Wang et al. 2021). Intriguingly, within *W.* sp. 2, China population has a 6% (dN) and a 4% (dS) higher substitution rate than *W.* sp. 2 from Taiwan (Table 2). Out of the 9,352 single-copy orthologs between the *Wiebesia* species, 643 have signatures of selection (dN/dS ratio > 1), which turned out to be enriched in gene ontologies (GOs) associated with serine-type endopeptidase and respiratory chain complex IV (Table 3).

**Table 2:** Estimation of nonsynonymous and synonymous substitutions, dN, and dS in each *Wiebesia* population accumulated since the divergence of *W.* sp 2 and *W.* sp. 3

	Nonsynonymous substitutions	Synonymous substitutions	dN (× 10^−3^)	dS (× 10^−3^)
**sp. 3**	8392.4^a^	17,347.4^a^	0.88 (0.93 ± 1.67)	5.89 (6.17 ± 8.38)
**sp. 2 (Taiwan)**	10,930.9^b^	20,754.9^b^	1.15 (1.28 ± 4.86)	7.05 (7.48 ± 15.16)
**sp. 2 (China)**	11,653.5	21,717.7	1.23 (1.37 ± 2.57)	7.34 (7.78 ± 10.32)

dN was calculated as the sum of nonsynonymous substitutions across all genes divided by the sum of nonsynonymous sites across genes.

dS was calculated as the sum of synonymous substitutions across all genes divided by the sum of synonymous sites across genes.

Values in parentheses are the mean per-gene dN and dS, with standard deviations.

^a^The number of mutations is significantly lower than that observed in sp. 2 from Taiwan.

^b^The number of mutations is significantly lower than that observed in sp.2 from China. *P* < 10^−3^, Tajima's one-degree-of-freedom (1D) test (Tajima 1993).

**Table 3:** Gene ontologies enriched in dN/dS > 1 dataset

GO term	GO ID	FDR
Component		
Respiratory chain complex IV	0045277	0.0031
Process		
Mitochondrial electron transport, cytochrome c to oxygen	0006123	0.0075
Proteolysis	0006508	7.60E−07
Function		
Cytochrome c oxidase activity	0004129	0.0031
Serine-type endopeptidase activity	0004252	9.47E−21

All GOs significant at 0.05 FDR level were shown.

### The Two *Wiebesia* Species Are Unequally Divergent in Host Specificity

**Fig. 3.**
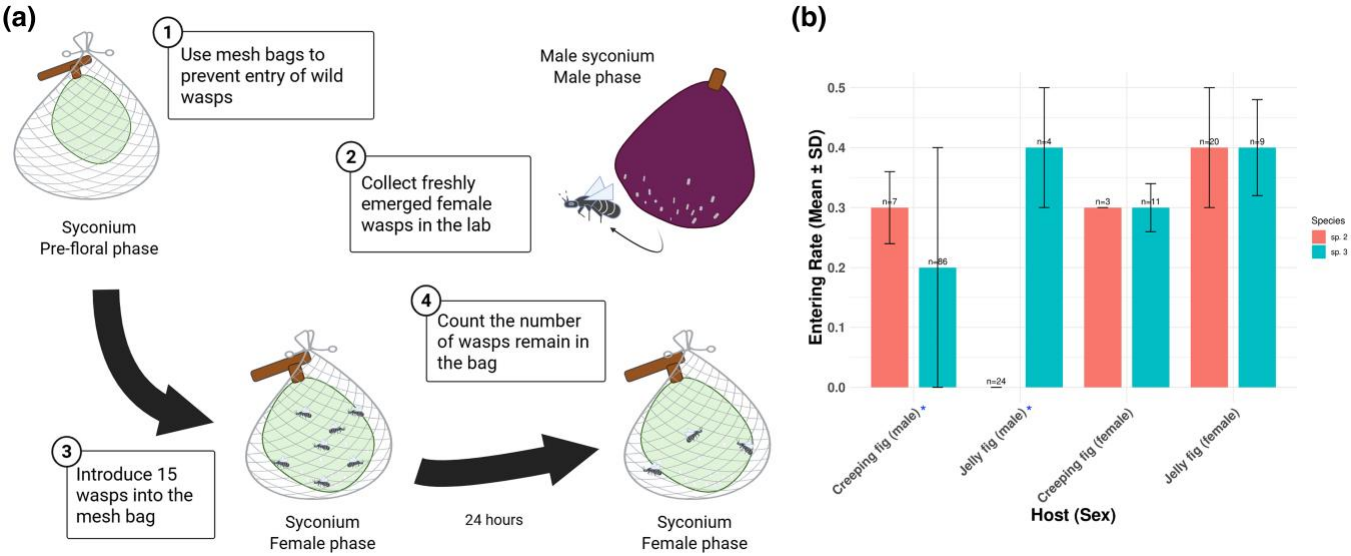
Setting and results of wasp introduction experiment. The experiment was conducted using eight distinct setups, incorporating combinations of two *Wiebesia* species, two host fig varieties, and two syconium types (male and female). a) The experiment was conducted in the field. Freshly emerged female wasps were captured and introduced to female phase syconia. The introduction was conducted between 11:00 and 13:00 when the wasps were more active. We count the number of remaining wasps after 24 h to estimate the entering rate. b) The results of introduction experiment. There are significant differences in the entering rates of male syconia between *Wiebesia* species (*t*-test, *P* < 10^−16^). Created with BioRender.com/8y8w2ot.

Both *Wiebesia* species showed clear differences in host attraction when exposed to the alternative host variety. Behavioral experiments on the two *Wiebesia* species revealed that when each species was introduced to their alternative host variety's male and female receptive fig syconia, they showed differential responses compared to their natural host variety controls (Fig. 3a and b). *W.* sp. 3 sporadically entered the male syconia of creeping figs (Table 4). However, it has a notably lower and less consistent entering rate (0.2 ± 0.2) compared to the natural pollinator *W.* sp. 2, which enters at a steadier and higher rate (0.3 ± 0.06, *P* < 10^−16^). In contrast, *W.* sp. 2 never entered the male syconia of jelly figs in all 24 repeated experiments. These results indicate partial host specificity and asymmetric host attraction performance between the two closely related fig wasps.

**Table 4:** Results of host attraction experiment

Fig host (sex)	*Wiebesia* species	Trials (*n*)	Entering rate	*P* value^a^
Creeping fig (male)	sp. 2	7	0.3±0.06	<10^−16^
	sp. 3	86	0.2±0.2	
Jelly fig (male)	sp. 2	24	0.0±0.0	<10^−16^
	sp. 3	4	0.4±0.1	
Creeping fig (female)	sp. 2	3	0.3±0.0	1
	sp. 3	11	0.3±0.04	
Jelly fig (female)	sp. 2	20	0.4±0.1	1
	sp. 3	9	0.4±0.08	

Entering rate: mean (± SD) proportion entering within 24 h.

^a^Two-tailed *t*-test.

### Divergence of Olfactory Expression Profiles

Comparative transcriptomic analyses of adult females of *W.* sp. 2 and *W.* sp. 3 (Figs. S4 and S5) revealed substantial interspecific differences in gene expression. Among 8,701 expressed genes, 432 were differentially expressed between the two species. GO enrichment analysis indicated that these genes are mainly associated with olfaction, cellular signal transduction, serine-type endopeptidase activity, and cuticle structure (Table 5). Chemosensory genes, which were differentially expressed in the two *Wiebesia* species, appeared to be single copy in *W.* sp. 2 and *W.* sp. 3, as well as the model fig wasp species *Ceratosolen solmsi*. Notably, *OBP11*, which detects repellent scent signals in *W.* sp. 2 (Wang et al. 2021), was significantly downregulated in *W.* sp. 3 (Table S6). Additionally, among chemosensory genes, ORs not only showed differential expression between species but also had elevated overall dN/dS ratios, with a significantly higher proportion of genes exhibiting a dN/dS ratio > 1 compared to the genome-wide background (Table S7).

**Table 5:** Gene ontologies enriched in cross-species differential expressed gene set

GO term	GO ID	FDR
Component		
Extracellular region	5576	8.75E−06
Process		
Sensory perception of smell	7608	1.66E−05
G protein-coupled receptor signaling pathway, coupled to cyclic nucleotide second messenger	7187	0.0015
Function		
Serine-type endopeptidase activity	4252	1.66E−05
Ion channel activity	5216	4.74E−05
OR activity	4984	3.72E−04
Odorant binding	5549	4.95E-04
Ion-gated channel activity	22,839	0.0015
Structural constituent of cuticle	42,302	0.0231

### Lineage-Specific Expansions and Reductions of OR Family

**Fig. 4.**
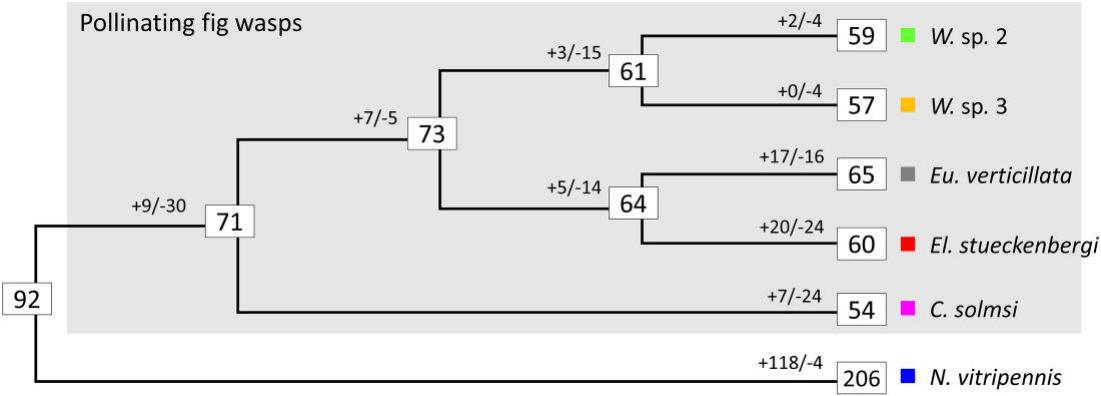
Maximum likelihood phylogeny of studied fig wasps and OR family size evolution. Numbers inside the boxes denote the size of ORs at the nodes and leaves. Numbers with a plus or minus sign before the boxes denote gene gain and loss along the branch. Abbreviations: *W*, *Wiebesia*; *Eu*, *Eupristina*; *El*, *Elisabethiella*; *C*, *Ceratosolen*; *N*, *Nasonia*.

To better understand how codivergence with host scents shapes the evolution of OR genes of pollinating fig wasps on a broader evolutionary scale, we expanded our samples to include fig wasp from another dioecious lineage, *C. solmsi*, and two monoecious fig wasp species, *Elisabethiella stueckenbergi* and *Eupristina verticillata*. Thus, our comparisons covered lineages from different host sexual systems, facilitating insights into OR family size evolution (Fig. 4). While the number of OR repertoires was largely conserved among all studied fig wasps, independent gains and losses of OR repertoires were found in each major lineage of pollinating fig wasps (Fig. 4).

**Fig. 5.**
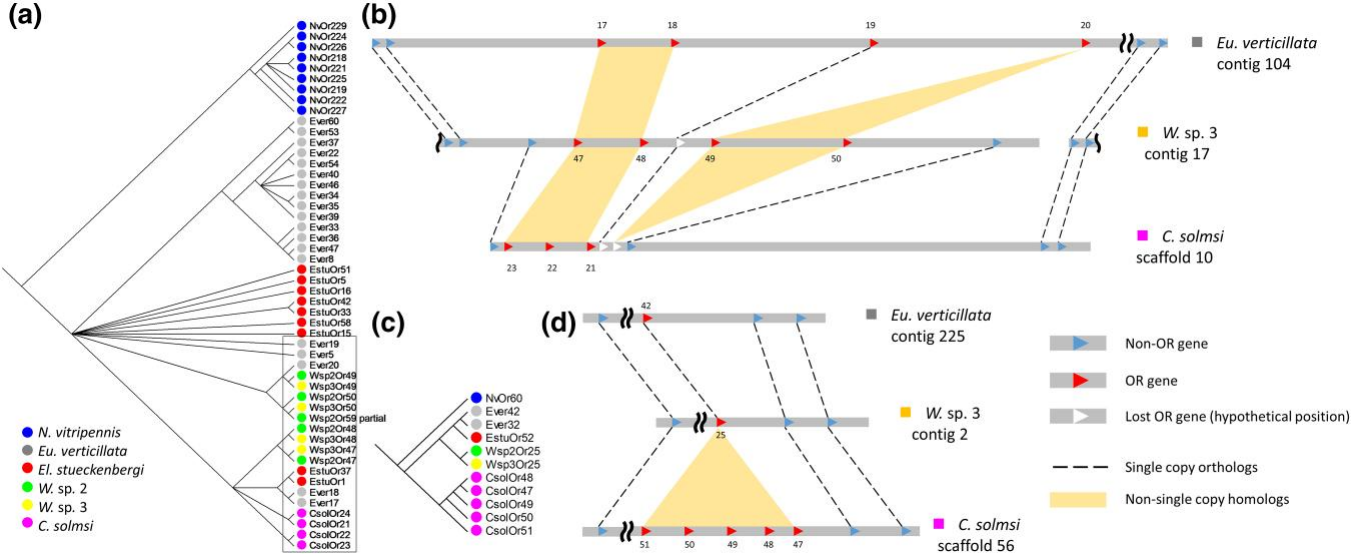
Examples of OR gene expansions due to tandem duplications in fig wasps. a) Subfamily Z of OR. Noting that all studied species experienced gene expansions, including the outgroup parasitoid wasp *N. vitripennis*. The synteny of the clade in square is shown in (b). Nodes with less than 50% bootstrap support were collapsed into polytomy. b) Local synteny of part of subfamily Z shows the dynamic nature of lineage-specific gene duplications and losses. *E. stueckenbergi* is excluded in the analysis because the OR annotation was derived from transcriptome. c and d) Tandem duplications resulted in a *C. solmsi*-specific expansion.

An in-depth examination of OR gene tree (Fig. S6; Fig. 5) revealed details of lineage-specific gene gains and losses. Notably, the subfamily Z has independently expanded in all four studied fig wasp genera (Fig. 4a). Other scattered lineage-specific expansions can be found across the gene tree (eg Fig. 4c; also see Fig. S6). Further syntenic comparisons indicated that these expansions are due to multiple local tandem repetitive arrays (Fig. 4b and d). Collectively, these data indicate that OR genes in pollinating fig wasps have evolved dynamically, with tandem duplications contributing to the emergence of novel OR genes.

### Accelerated Evolution in Duplicated ORs

To test whether the duplicated ORs experienced different selective pressure, we applied codon-based branch site selection analysis. We first assessed the quality of the annotated ORs by predicting the number of transmembrane domains present in these genes. Only species with an average number of TDs above five were used to ensure adequate information (Table S8). These included *C. solmsi* and the two *Wiebesia* species. The ORs from these three species were divided into two groups: simple orthogroups and complex orthogroups (Fig. S7). The former consist of single-copy orthologs from the three species, while the latter contain monophyletic gene trees that include duplicated paralogs. Notably, five out of the 41 branches in complex orthogroups exhibited signatures of positive selection, a rate significantly higher than the two out of 87 branches in simple orthogroups (*P* < 0.05).

## Discussion

In this study, we investigated the codiversification genomic landscape in two closely related *Wiebesia* fig wasp species. By combining genomic, transcriptomic, and behavioral data, we revealed the genetic signatures underlying ecological specialization and olfactory coevolution in these pollinating wasps. Our findings highlight the strong influence of host life histories and ecological conditions on the evolutionary trajectories of their pollinators, demonstrating how tightly linked life cycles shape adaptation and diversification.

### The Divergence and Ecological Differentiation of *Wiebesia* spp. and Their Fig Hosts

Our demographic modeling indicates that the common ancestor of *W.* sp. 2 and *W.* sp. 3 began diverging 547,000 years ago (95% CI: 178,825 to 645,899) during the Middle Pleistocene. This likely corresponds to the colonization of Taiwan by the ancestral fig–fig wasp lineage from mainland East Asia (Fig. 2b1). Effective population size estimates also reflect differences in their distribution ranges: The Ne of the widely distributed *W*. sp. 2 is about twice that of the Taiwan-endemic *W*. sp. 3 (Fig. 2a). We note that our nucleotide-diversity estimates may be conservative because pooled-seq from foundresses entering a single syconium may not capture the full among-syconia variation expected under heterogeneous foundress numbers. Nevertheless, two lines of evidence suggest that our estimates are still within a reasonable range. First, genetic diversity in the two species is approximately half of that observed in *Drosophila melanogaster* (Chen et al. 2024). Because fruit fly has a much larger population size and broader geographic distribution than these fig wasps, higher diversity in *D. melanogaster* is expected. Thus, the slightly lower diversity in fig wasps is expected and suggests that we capture substantial genetic variation. Second, a recent study of *Pegoscapus hoffmeyeri* sp. A sequenced eight female offspring from independent syconia and reported nucleotide diversity (π) on the order of 10^−4^ to 10^−3.5^, roughly an order of magnitude lower than that observed in *W.* sp. 2 and *W.* sp. 3 (Zhao et al. 2025). Together, these comparisons suggest that, although our estimates may be conservative, they likely provide a reasonable approximation of genome-wide diversity in *Wiebesia*. Future work using whole-genome sequencing of multiple individuals from multiple syconia (and ideally from different localities) would provide a more complete estimate of diversity and stronger population genomic inference.

Migration between the two species ceased around 80,000 years ago (95% CI: 26,390 to 168, 648), coinciding with the beginning of the Last Glacial Period (115,000 to 11,700 years ago) (Fig. 2b2). During this period, the jelly fig–*W.* sp. 3 likely remained confined to Taiwan, whereas their mainland siblings retreated to warmer southern refugia. This scenario is consistent with the retreat of creeping fig-favored biome in the Last Glacial Maximum, inferred from ancient pollen records (Yu et al. 2000; Harrison et al. 2001). Migration resumed around 10,000 years ago (95% CI: 5,514 to 12,687) and persists to the present day (Fig. 2a). This coincides with the postglacial expansion of creeping fig across East Asia and its recolonization of lowland Taiwan (Fig. 2b3) (Wang et al. 2013).

Previous studies using genetic markers have linked Pleistocene climate oscillations to *Wiebesia* spp. distribution and diversification (Chen et al. 2012; Lo and Wang 2021). Using genome-wide data, our analyses refine this scenario by supporting a later divergence between the two focal lineages and by clarifying why the jelly fig–*W.* sp. 3 association is now concentrated at ∼500 to 2,000 m in Taiwan. A parsimonious explanation is that their ancestors were adapted to an environment 6 to 7 °C cooler during the Last Glacial Period (Lin 1963) and that this cold-associated adaptation subsequently translated into present-day elevational restriction as temperatures warmed during the Holocene. Consistent with this, our earlier work demonstrated longer survival of *W*. sp. 3 under low temperatures and signatures of positive selection in mitochondrial COX1 relative to *W.* sp. 2 (Wang et al. 2013). Here, we extend that finding by detecting positive selection in nuclear-encoded mitochondrial complex IV genes along the *W.* sp. 3 lineage. Because COX1 is itself a core subunit of complex IV, the concordant signal across mitochondrial and nuclear components suggests that selection targeted oxidative phosphorylation more broadly, potentially helping maintain electron-transport efficiency, and thus ATP supply, under cold stress in the jelly fig habitat.

Beyond respiratory-chain genes, we also observed enrichment of selection signals in proteolysis and serine-type endopeptidase activity, pointing to a wider metabolic footprint of divergence. This is particularly relevant because larval development occurs entirely within the syconium, where nutrient composition and microenvironment can differ between host varieties and across seasons. In this context, adaptive shifts in digestive enzymes could reflect optimization of nutrient acquisition and allocation, which would plausibly interact with mitochondrial energy metabolism to support growth, development, and adult performance in cooler environments. It is important to note that “metabolic adaptation” need not imply a single causal axis. Instead, our data suggest repeated selection on pathways involved in energy production and resource processing in *W.* sp. 3. Nevertheless, linking these genomic signatures to specific performance traits will require further targeted functional validation.

Host phenology provides a complementary ecological mechanism that can connect metabolic evolution with lineage-specific evolutionary rates. Depending on geographical location, male creeping figs typically have three or four rounds of syconium cycle per year (Ho et al. 1998), whereas male jelly figs have only two (Shih 1988). Because each cycle is synchronized such that syconia become receptive when wasps from the previous crop emerge, fig wasp generation time is constrained by host phenology (Bronstein and Patel 1992). Accordingly, we observed a significantly higher substitution rate in *W.* sp. 2 than in *W.* sp. 3 (Table 3), consistent with generation-time expectations (Li et al. 1996). Temperature could further amplify this difference because developmental time is temperature sensitive and warmer conditions may increase mutation rates in ectothermic insects (Waldvogel and Pfenninger 2021), which may help explain why substitution rates were highest in the mainland *W.* sp. 2 population. The lower substitution rate in the Taiwan *W.* sp. 2 population may reflect introgression from *W.* sp. 3, consistent with our demographic analyses, although this inference should be interpreted cautiously because rate estimates can be influenced by multiple demographic and genomic factors.

Taken together, our demographic, selection, and evolutionary rate analyses support a scenario in which isolation of the jelly fig–*W.* sp. 3 from mainland relatives during the Last Glacial Period led to local adaptation to colder environments. These adaptations later manifested as ecological niche partitioning under Holocene climate. The cold-adapted nature of *W.* sp. 3 also suggests more vulnerability to ongoing climate change. While wild jelly fig populations may shift to higher elevations, cultivated jelly fig, which mainly grown in lowland regions, may be even more strongly affected.

### Genetic Basis of Host Specificity in the Two *Wiebesia* Species

Olfactory coadaptation is arguably the single most important trait for the wasps in maintaining host–pollinator specificity in fig–fig wasp mutualism (Janzen 1979; Hossaert-McKey et al. 2010). Previous studies have shown that the two *F. pumila* varieties have different floral volatile profiles (Chen and Wu 2010; Chen et al. 2016). Therefore, it is reasonable to expect differentiation in chemosensory genes between *Wiebesia* species. Our gene expression and evolutionary analyses revealed that chemosensory genes are differentially expressed between *Wiebesia* species (Table S6) and exhibit signs of adaptive evolution (Table S7), suggesting their contribution in shaping host specificity. The behavioral experiments demonstrated host specificity in *W.* sp. 2 and *W.* sp. 3, although *W.* sp. 3 can also respond to the male syconia of creeping fig, a phenomenon that was noted also in field sampling (Chen et al. 2012; Wang et al. 2013). These findings highlight the role of olfactory adaptations in driving the evolutionary divergence of pollinators in response to host-specific chemical cues.

An unexpected discovery in our behavioral experiment is that both species are drawn to the female syconia of the alternative host. This is particularly intriguing for *W.* sp. 2, as it suggests that this species can distinguish between male and female syconia of the jelly fig. This finding challenges the “no preference hypothesis,” which states that syconia of both sexes mimic each other (Grafen and Godfray 1991). Such similar chemical profiles between sexes have been observed in the syconia of creeping fig (Wang et al. 2021). However, it is also possible that male and female syconia are not chemically identical but appear indistinguishable only to their coevolving pollinators. Furthermore, geographical isolation plays a crucial role in the divergence of the two host–pollinator pairs, reducing the likelihood of the wasps encountering the chemical signals from the other variety during codivergence. Consequently, there was less active selection against cross-variety entry, which accounts for the presence of this behavior in both *W.* sp. 2 and sp. 3.

Among the chemosensory genes, *OBP11* is an interesting candidate that may explain the phenotypic divergence between the two wasps. We found a significantly higher expression of *OBP11* in *W.* sp. 2 compared to *W.* sp. 3. *Wiebesia* sp. 2 uses *OBP11* to detect the repellent molecule nonanal emitted from creeping fig (Wang et al. 2021). Receptive stage male syconia of jelly fig (the host of *W.* sp. 3) emitted relatively higher amount of nonanal than that of creeping fig (Chen and Wu 2010; Chen et al. 2016). We therefore speculate that the higher expression of *OBP11* in *W.* sp. 2 leads to a higher sensitivity to the repelling signal of nonanal than in *W.* sp. 3. This may explain why *W.* sp. 2 is less attracted by jelly fig, while *W*. sp. 3, though showing a preference, is more flexible in its attraction to both varieties.

### Dynamics of ORs and Implications on Long-term Fig–Fig Wasp Coevolution

The mass reduction in chemosensory receptor gene numbers among fig wasps has been attributed as a consequence of their mutualistic association with fig trees (Xiao et al. 2013, 2021). On the other hand, a recent transcriptome study has demonstrated that while the total number of OR genes is conserved and highly reduced in fig wasps, distinct lineages exhibit varied patterns of gains and losses (Yu et al. 2023). Our genome-based analysis confirmed this finding. Additionally, we showed that many novel OR genes arose from local tandem duplications, with these duplicated ORs exhibiting a higher frequency of positive selection signatures compared to copy-number-conserved counterparts, indicating functional divergence of duplicate genes.

Expansion of chemosensory receptors has been linked to various olfaction-related adaptations in insects (Engsontia et al. 2014; Harrison et al. 2018). A famous example in hymenoptera is the independent expansions of the OR 9-exon subfamily in several eusocial lineages (Engsontia et al. 2015; Brand and Ramírez 2017; Legan et al. 2021). It is yet not surprising that pollinating fig wasps followed the same evolutionary path. Expansions were most prominent in one section of the subfamily Z (sister to NvOr218-229 of the parasitoid wasp *Nasonia vitripennis*), which all four of our studied genera had genus-specific expansions, making this section of subfamily Z one likely candidate responsible for host scent detection. Subfamily Z contains only a singleton gene in most hymenopterans, including sawfly *Cephus cinctus* (CcinOr52) (Robertson et al. 2018), ants, and bees (PbarOr152, BterOr110, and AmelOr141) (Zhou et al. 2015), but is greatly expanded in parasitoid wasps, such as *N. vitripennis* (NvOr218-236). We therefore speculate that subfamily Z is of functional use in parasitoid wasps, and OR genes from it were coopted to detect host scents in the transition from parasitoid to nursery pollinating lifestyle. Taken together, adaptive gene duplication in ORs, such as the one observed in subfamily Z, likely played a crucial role in the coevolution of fig floral scents and wasp olfactory recognition.

## Conclusion

Through multiomic and behavioral analyses of pollinating wasps, we detected genome-wide signatures of positive selection in *W.* sp. 3 associated with energy production and metabolism, suggesting adaptations to colder, mid-elevation environments. We also observed a significantly lower substitution rate in *W.* sp. 3 relative to *W.* sp. 2, consistent with longer generation times linked to their high-elevation, slower-growing host, the jelly fig. In addition, divergence in chemosensory gene expression and adaptive expansions in OR genes point to mechanisms of coadaptation to host floral scents.

Together, these findings illustrate that because fig wasps are tightly synchronized with their hosts’ life cycles, the life history traits and habitat preferences of fig trees can directly shape the evolutionary trajectories of their associated pollinators, leaving distinct genomic signatures. Our results therefore contribute to a broader understanding of coevolutionary dynamics by uncovering the genomic processes underlying codiversification.

Future research should focus on exploring the full cophylogeny, including the two host varieties, as well as other copollinators, such as *W.* sp. 1 (Chen et al. 2012). While our study integrates multiomic and behavioral approaches, more detailed behavioral assays (e.g. Y-tube choice tests), biochemical analysis of syconium volatile profiles, and functional genomic studies of wasps are necessary to bridge the gap between molecular and gross phenotypes.

## Materials and Methods

### Biological Materials and Sequencing

Genomic DNA was extracted from 30 pooled male individuals of each species from a single syconium using Purgene kit (Qiagen, USA). Whole-body RNA from five stages (adult female, adult male, pupal female, pupal male, and larva) was extracted using TRIzol (Thermo Fisher, USA). Library preparation and sequencing were performed by Novogene, Beijing, China. Long-read sequencing of *W.* sp. 3 was done on a PacBio Sequel platform. DNA and RNA libraries that had insert size around 300 to 500 bp were prepared for *W.* sp. 3 and *W.* sp. 2 and sequenced on Illumina Hiseq 4000 platform. The libraries and sample information are summarized in Table S1.

### Genome Assembly and Annotation

Short genomic reads were quality trimmed by Trimmomatic v0.36 (Bolger et al. 2014). Quality assessment was done by FastQC v0.10.1 (Andrews 2010). Jellyfish v2.2.6 (Marçais and Kingsford 2011) was used to estimate genome size from kmer. An initial genome was assembled from long reads by wtdbg v1.1.006 (Ruan and Li 2020) and evaluated by blobtools v1.1 (Laetsch and Blaxter 2017). Both short and long reads were filtered by blobtools to remove contaminations. Filtered long reads were assembled by wtdbg and polished with arrow and pilon v1.2 (Walker et al. 2014). By the time of the analysis, the *W.* sp. 2 genome from population in China has not been released. Therefore, the assembled *W* sp. 3 genome served as a backbone for the reference-based assembly of *W.* sp. 2. Short reads of *W.* sp. 2 were mapped against *W.* sp. 3 genome using BWA mem (Li and Durbin 2009). Variants from alignment file were called using samtools v1.2 (Li 2011). Consensus sequence was generated by bcftools v1.3 (Li 2011) in genomic regions that have a coverage between one-third and two times the mean coverage; regions that have coverage outside the range were substituted by Ns. Pilon was applied iteratively on the *W.* sp. 2 genome to update the genome based on information from short reads. Quast v4.6.3 (Gurevich et al. 2013) was used to calculate assembly statistics; busco v3 (Simão et al. 2015) was used to evaluate the completeness based on Hymenoptera odb9 dataset (Zdobnov et al. 2017). Foreign contigs detected by blobtools were excluded before annotation. D-Genies server (Cabanettes and Klopp 2018) was used to perform dotplot analysis on the assembled *W.* sp. 3 genome and the published *W.* sp. 2 (China) genome.

The two assembled genomes were annotated using the same pipeline. Annotation of repetitive DNAs based on homology was done by RepeatMasker v4.0 (Tarailo-Graovac and Chen 2009) with data from Repbase (Bao et al. 2015) and the ones predicted by RepeatModeler v1.0.1 (Tarailo-Graovac and Chen 2009). Repeat-masked genomes were created by bamtools v2.5.1 (Barnett et al. 2011) and were used in ab initio gene predictions. Gene predictions based on transcriptome, protein homology, and ab initio methods were loaded to Evidencemodeler v1.1.1 (EVM) (Haas et al. 2008) to generate consensus gene sets. Consensus gene sets were manually curated using Web Apollo v2.1.0 (Lee et al. 2013). Blast2GO v5.2.1 (Gotz et al. 2008), which integrated blast and interproscan (Jones et al. 2014) annotation results, was used for functional and GO annotation. Online server of BlastKOALA (Kanehisa et al. 2016) was used to map the gene sets to Kyoto Encyclopedia of Genes and Genomes database (Kanehisa and Goto 2000).

### Demographic Analysis

PPalign module in the PoolParty pipeline was used to call single nucleotide polymorphisms (SNPs) from short-read libraries (Micheletti and Narum 2018). Reads were first trimmed by BBduk to remove adapters, contaminants, low-quality reads (<20), and reads less than 25 bp in length (Bushnell 2014). Trimmed reads were aligned to *W.* sp3 assembly with a minimum mapping quality of 20 using the BWA-MEM (Li and Durbin 2009). Duplicate reads were removed by SAMBLASTER (Faust and Hall 2014), and reads with mapping quality less than five were filtered in SAMtools before being converted to bam files (Li 2011). SNPs were called in BCFtools with a SNP quality threshold of 20. After applying the initial filter criteria, the variant dataset was limited to biallelic sites using VCFtools (Danecek et al. 2011).

The folded site frequency spectrum (SFS) for all populations were generated from the final variant calls (vcf files) using easySFS (https://github.com/isaacovercast/easySFS) (Gutenkunst et al. 2009). Demographic parameters were estimated from the joint SFS using coalescent simulations (fastsimcoal2) (Excoffier et al. 2021). The likelihood of the observed SFS under six complex demographic scenarios was calculated. Each model was run 100 replicated times considering 100,000 to 250,000 simulations for the calculation of the composite likelihood. We used an information-theoretic model selection approach based on the Akaike's information criterion (AIC) to determine the probability of each model given the observed data. The mutation rate of 2.8 × 10^−8^ per site per year from *N. vitripennis*, another member of the superfamily Chalcidoidea, was applied (Xu et al. 2021).

### Phylogenetic Analyses

The published transcriptome reads of *E. stueckenbergi* (SRR1502962) were de novo assembled with Trinity v2.8.4 (Haas et al. 2013). Transcript isoforms were filtered so that only the longest isoform was left as a representative of each gene. Protein sequences of *C. solmsi*, *E. verticillata*, and *N. vitripennis* (Pteromalidae) were downloaded from publications (Zhou et al. 2015; Zhang et al. 2020). A total of 2,684 busco genes that were single copy in all six species were used to reconstruct phylogeny of fig wasps. The genes were aligned with MAFFT v6 (Katoh and Standley 2013), filtered with trimal 2 (Capella-Gutiérrez et al. 2009) using default parameters, and then concatenated. Maximum likelihood tree of the concatenated busco genes was constructed using raxml-ng (Stamatakis 2014) with 100 bootstraps.

### Evolutionary Rates and Signatures of Selection Between *Wiebesia* Species

Orthology between the four protein-coding gene annotations, *W.* sp. 3, *W.* sp. 2 (Taiwan), *W.* sp. 2 (China), and *C. solmsi*, were determined using proteinortho v5.16 (Lechner et al. 2011). One-to-one orthologs were aligned by codon using PRANK v.14 (Löytynoja 2014) and filtered with Gblocks 0.91b (Castresana 2000). Branch-specific dN and dS, as well as the numbers of synonymous and nonsynonymous substitutions, were estimated using codeml free ratio model from paml v4.9 (Yang 2007). We then summed synonymous and nonsynonymous substitutions across genes and applied Tajima's one-degree-of-freedom (1D) test to evaluate whether *W.* sp. 3 and *W.* sp. 2 have similar mutation rates (Tajima 1993). GO enrichment analysis using Blast2GO was performed on the sets of genes with a dN/dS ratio larger than 1 with a false discovery rate (FDR) cutoff 0.05 (Benjamini and Hochberg 1995).

### Fig Wasp Host Attraction Experiment

Eight combinations of lineage and sex were used for behavioral experiments (two wasp species × two syconia sexes × two host varieties). Each combination has a minimum of three experimental replicates (Table 5). To avoid influence from wild wasps, all the syconia used for entry experiment were sealed with mesh bag in prefloral phase. To collect female wasps, near D phase syconia were brought back to the laboratory, where the emerging wasps were collected with mesh bags. These freshly emerged female wasps were then brought to the field for behavioral experiments. Fifteen female adult wasps were introduced inside the mesh bag of each receptive syconium. The number of entry wasps was calculated 24 h after the introduction. Entering rate is calculated as the number of entry wasps/15. The *t*-test was used for statistical testing between combinations.

### Cross-Species Differential Expression Analysis of the *Wiebesia* Species

Near D phase syconia were brought back to the laboratory, and freshly emerged female wasps were collected for sequencing. Four biological replicates of adult female transcriptome were sequenced for each species. Transcript per million reads (TPM) for each gene was calculated with the Perl script “align_and_estimate_abundance.pl” provided by Trinity (Haas et al. 2013) for each library. Kallisto v0.44 (Bray et al. 2016) was used for abundance estimation. TPM values were adjusted by ortholog length using the “scaledTPM” method from Bioconductor package “tximport” (Soneson et al. 2015). Cross-sample normalization was then applied on the adjusted TPM values using the trimmed Mean of M-values method (Robinson et al. 2010). Biological replicate validation was done by principal component analysis and hierarchical clustering using R. Differential expression analysis was performed on all single-copy orthologs that are expressed in at least two libraries using the Bioconductor package edgeR (Robinson et al. 2010) with minimum log fold change set to 4 and a FDR cutoff of 0.05. GO enrichment analysis of differentially expressed genes was done by Blast2GO based on annotation of *W.* sp. 3, with a FDR cutoff of 0.05.

### Manual Annotation of ORs and Evolutionary Analysis

Amino acid sequences of Hymenoptera ORs (Zhou et al. 2015) were downloaded as query dataset. Manual annotation of chemosensory genes in the four fig wasp species, *W.* sp. 3, *W.* sp. 2, *E. stueckenbergi*, and *E. verticillata*, was done with two approaches. First, we blasted the annotated gene sets against the query set. Second, we conducted homology-based gene model prediction using GenBlastA v1.0.4 (She et al. 2009), followed by refinement with GeneWise v2.2 (Birney et al. 2004) with an E-value cutoff of 10^−5^ to finalize the gene model based on query sequences. Results of the two approaches were manually combined under Web Apollo. Maximum likelihood phylogeny of fig wasp ORs was created by raxml-ng v1.0.2 and labeled using MEGA7 (Kumar et al. 2016). Orthology of genes was determined by both proteinortho and phylogenetic relationships. TMHMM v2.0 server (Krogh et al. 2001) was used to annotate transmembrane domain in the OR genes. Gene birth and death events were interpreted using Notung 2.9.1.5 (Chen et al. 2000), with a raxml-ng tree as input. The rearrange threshold for bootstrap values was set to 70. Tandem repeats were identified by manual inspecting the coordinates of the OR genes.

Both *E. stueckenbergi* and *E. verticillata* were excluded in cds-based analysis because of annotation quality. OR gene tree was divided into two types of orthogroups. Each orthogroup was consisted of at least three sequences from at least two species. A simple orthogroup consisted of single-copy orthologs from each of the three fig wasp species only, while a complex orthogroup contains paralogs. Each orthogroup was aligned by codon using PRANK. Low-quality sites were filtered by Gblocks. The filtered alignments were manually examined so that incomplete genes can be excluded. Branch-site test using aBSREL (Smith et al. 2015) from online server of Datamonkey (Weaver et al. 2018) was performed on all the subtrees to identify branches under positive selection. A custom R script was used to adjust for multiple testing using FDR and perform Fisher’s exact test to test whether positive selection occurred significantly differently between groups. The significance cutoff for FDR and Fisher’s exact test *P* value are both 0.05.

## Supplementary Material

evag080_Supplementary_Data

## Data Availability

Genomes and the raw reads used for this study can be found under the NCBI BioProject: PRJNA730931. Additional information including functional annotations, OR genes, and scripts for this study can be found at https://github.com/BaiweiLo/Wiebesia_wasp_genomes
